# Supplementary material for: Dkk1 as a Prognostic Marker for Neoadjuvant Chemotherapy Response in Breast Cancer Patients
Source: Cancers (Basel). 2024 Jan 18;16(2):419. doi: 10.3390/cancers16020419 (PMC10814026; doi:10.3390/cancers16020419)
Supplement: Supplementary file 1 [file cancers-16-00419-s001.zip › Supplementary Table S1.pdf]

Supplementary Table S1. Dkk1-IRS reduction scores in 68 matched cases

	**Core needle biopsy tissues**			**Mammary carcinoma tissues**			**Differencies in Dkk1 expression**	
**Case**	***Percentage of stained tumour cells**	**°Staining intensity**	**ΔImmuno-reactive score (IRS 1)**	***Percentage of stained tumour cells**	**°Staining intensity**	**ΔImmuno-reactive score (IRS 2)**	**IRS 1–IRS 2**	**†Reduction percentage (%)**
**1**	4	2	8	4	0	0	8	100
**2**	4	2	8	4	0	0	8	100
**3**	4	2	8	0	0	0	8	100
**4**	4	1	4	0	0	0	4	100
**5**	4	1	4	4	0	0	4	100
**6**	4	1	4	0	0	0	4	100
**7**	4	2	8	4	0	0	8	100
**8**	3	1	3	4	0	0	3	100
**9**	4	2	8	4	0	0	8	100
**10**	4	1	4	4	0	0	4	100
**11**	4	3	12	2	1	2	10	83
**12**	4	3	12	2	1	2	10	83
**13**	4	3	12	2	1	2	10	83
**14**	3	3	9	2	1	2	7	78
**15**	4	2	8	2	1	2	6	75
**16**	4	2	8	2	1	2	6	75
**17**	4	2	8	2	1	2	6	75
**18**	4	2	8	2	1	2	6	75
**19**	4	2	8	2	1	2	6	75
**20**	4	3	12	3	1	3	9	75
**21**	4	3	12	3	1	3	9	75
**22**	4	3	12	4	1	4	8	67
**23**	3	2	6	2	1	2	4	67
**24**	4	3	12	4	1	4	8	67
**25**	4	3	12	4	1	4	8	67
**26**	4	2	8	3	1	3	5	63
**27**	4	2	8	4	3	3	5	63
**28**	4	2	8	3	1	3	5	63
**29**	4	2	8	3	1	3	5	63
**30**	4	2	8	3	1	3	5	63
**31**	4	2	8	3	1	3	5	63
**32**	3	3	9	4	1	4	5	56
**33**	3	3	9	2	2	4	5	56
**34**	3	3	9	4	1	4	5	56
**35**	4	2	8	4	1	4	4	50
**36**	4	3	12	3	2	6	6	50
**37**	4	2	8	4	1	4	4	50
**38**	4	2	8	4	1	4	4	50
**39**	3	2	6	3	1	3	3	50
**40**	4	3	12	3	2	6	6	50

	Core needle biopsy tissues			Mammary carcinoma tissues			Differencies in Dkk1 expression	
**Case**	***Percentage of stained tumour cells**	**°Staining intensity**	**ᐞImmuno-reactive score (IRS 1)**	***Percentage of stained tumour cells**	**°Staining intensity**	**ᐞImmuno-reactive score (IRS 2)**	**IRS 1–IRS 2**	**‡Reduction percentage (%)**
**41**	4	2	8	4	1	4	4	50
**42**	4	2	8	4	1	4	4	50
**43**	3	3	9	3	2	6	3	33
**44**	4	3	12	4	2	8	4	33
**45**	4	3	12	4	2	8	4	33
**46**	3	2	6	4	1	4	2	33
**47**	4	3	12	4	2	8	4	33
**48**	4	3	12	4	2	8	4	33
**49**	4	3	12	4	2	8	4	33
**50**	4	3	12	4	2	8	4	33
**51**	4	3	12	4	2	8	4	33
**52**	4	3	12	4	2	8	4	33
**53**	4	3	12	4	2	8	4	33
**54**	4	1	4	3	1	3	1	25
**55**	4	2	8	2	3	6	2	25
**56**	3	3	9	4	2	8	1	11
**57**	4	2	8	4	2	8	0	0
**58**	4	2	8	4	2	8	0	0
**59**	4	2	8	4	2	8	0	0
**60**	4	1	4	4	1	4	0	0
**61**	3	1	3	3	1	3	0	0
**62**	4	3	12	4	3	12	0	0
**63**	4	2	8	4	2	8	0	0
**64**	4	3	12	4	3	12	0	0
**65**	4	2	8	4	2	8	0	0
**66**	4	1	4	4	2	8	-4	-100
**67**	2	1	2	4	1	4	-2	-100
**68**	3	1	3	4	2	8	-5	-167

*The percentage of stained tumour cells was divided into four categories: <10% of cells (Score 1), 10–50% of cells (Score 2), 51–80% of cells (Score 3), and >80% of cells (Score 4). °The intensity of staining was classified as negative (Score 0), weakly positive (Score 1), moderately positive (Score 2), or strongly positive (Score 3). ᐞIRS was calculated by multiplying the percentage of stained tumour cells by the intensity of the staining. IRS values ranged from 0 to 12. Dkk1 expression was graded as either negative (IRS=0–2), weak (IRS=3–4), moderate (IRS=6–8), or strong (IRS=9–12). ‡Dkk1 reduction percentage was calculated using the following formula: Dkk1 reduction percentage = [(Dkk1-IRS in core needle biopsy tissue-Dkk1-IRS in mammary carcinoma tissue)x100] / Dkk1-IRS in core needle biopsy tissue.
