# Supplementary material for: Dkk1 as a Prognostic Marker for Neoadjuvant Chemotherapy Response in Breast Cancer Patients
Source: Cancers (Basel). 2024 Jan 18;16(2):419. doi: 10.3390/cancers16020419 (PMC10814026; doi:10.3390/cancers16020419)
Supplement: Supplementary file 1 [file cancers-16-00419-s001.zip › Supplementary Table S2.pdf]

Supplementary Table S2. The clinicopathological findings of women cases with increased Dkk1-IRSs

Case	Age (Years)	BMI	TNM staging at time of diagnosis			G status	Ki-67 Index (%)	Histological subtype	TNM staging at time of operation			*Therapy completed	R	OS	Dkk1-IRS Core-needle Biopsy tissue	Dkk1-IRS Mammary carcinoma tissue	Increasment percentage (%)
			T	N	M				T	N	M						
1	45	37	3	1	0	2	10	Luminal A	4	0	1	Yes	1	Died	3	8	167
2	65	24	2	3	0	3	80	TNBC	4	2	1	No	1	Died	4	8	100
3	42	32	2	0	0	3	30	TNBC	1	3	1	Yes	2	Died	2	4	100

*All three patients recieved NACH inclusing Taxanes (Paclitaxel or Docetaxel), Epirubicin, and Cyclophosphamide. BMI: Body mass index. R: Regression grade according to Sinn. OS: Overall survival status.
