# Supplementary material for: Dkk1 as a Prognostic Marker for Neoadjuvant Chemotherapy Response in Breast Cancer Patients
Source: Cancers (Basel). 2024 Jan 18;16(2):419. doi: 10.3390/cancers16020419 (PMC10814026; doi:10.3390/cancers16020419)
Supplement: Supplementary file 1 [file cancers-16-00419-s001.zip › Supplementary Table S3.pdf]

Supplementary Table S3. NACT protocols of 50 patients with completed treatment courses

Nr.	Therapy	Dkk1-IRS reduction (%)
**1**	4xEC (Epirubicin 90 mg/m²+Cyclophoshamide 600 mg/m² i.v./3 weeks), followed with 12xPaclitaxel (80 mg/m² i.v./week)	33
**2**	4xEC (Epirubicin 90 mg/m²+Cyclophoshamide 600 mg/m² i.v./3 weeks), followed with 12xPaclitaxel (80 mg/m² i.v./week)	33
**3**	4xEC (Epirubicin 90 mg/m²+Cyclophoshamide 600 mg/m² i.v./3 weeks), followed with 12xPaclitaxel (80 mg/m² i.v./week)	83
**4**	4xEC (Epirubicin 90 mg/m²+Cyclophoshamide 600 mg/m² i.v./3 weeks), followed with 12xPaclitaxel (80 mg/m² i.v./week)	100
**5**	4xEC (Epirubicin 90 mg/m²+Cyclophoshamide 600 mg/m² i.v./3 weeks), followed with 12xPaclitaxel (80 mg/m² i.v./week)	33
**6**	4xEC (Epirubicin 90 mg/m²+Cyclophoshamide 600 mg/m² i.v./3 weeks), followed with 12xPaclitaxel (80 mg/m² i.v./week)	0
**7**	4xEC (Epirubicin 90 mg/m²+Cyclophoshamide 600 mg/m² i.v./3 weeks), followed with 12xPaclitaxel (80 mg/m² i.v./week)	0
**8**	4xEC (Epirubicin 90 mg/m²+Cyclophoshamide 600 mg/m² i.v./3 weeks), followed with 12xPaclitaxel (80 mg/m² i.v./week)	67
**9**	4xEC (Epirubicin 90 mg/m²+Cyclophoshamide 600 mg/m² i.v./3 weeks), followed with 12xPaclitaxel (80 mg/m² i.v./week)	50
**10**	4xEC (Epirubicin 90 mg/m²+Cyclophoshamide 600 mg/m² i.v./3 weeks), followed with 12xPaclitaxel (80 mg/m² i.v./week)	63
**11**	4xEC (Epirubicin 90 mg/m²+Cyclophoshamide 600 mg/m² i.v./3 weeks), followed with 12xPaclitaxel (80 mg/m² i.v./week)	56
**12**	3xEC (Epirubicin 90 mg/m²+Cyclophoshamide 600 mg/m² i.v./3 weeks), followed with 9xPaclitaxel (80 mg/m² i.v./week)	63
**13**	4xEC (Epirubicin 90 mg/m²+Cyclophoshamide 600 mg/m² i.v./3 weeks), followed with 12xPaclitaxel (80 mg/m² i.v./week) +Carboplatin (AUC 1,5-2 i.v./week)	100
**14**	4xEC (Epirubicin 90 mg/m²+Cyclophoshamide 600 mg/m² i.v./3 weeks), followed with 12xPaclitaxel (80 mg/m² i.v./week) +Carboplatin (AUC 1,5-2 i.v./week)	63
**15**	4xEC (Epirubicin 90 mg/m²+Cyclophoshamide 600 mg/m² i.v./3 weeks), followed with 12xPaclitaxel (80 mg/m² i.v./week) +Carboplatin (AUC 1,5-2 i.v./week)	100
**16**	4xEC (Epirubicin 90 mg/m²+Cyclophoshamide 600 mg/m² i.v./3 weeks), followed with 12xPaclitaxel (80 mg/m² i.v./week)+(Trastusumab 6mg/kg+420 mg Pertuzumab i.v./3 weeks)	0
**17**	4xEC (Epirubicin 90 mg/m²+Cyclophoshamide 600 mg/m² i.v./3 weeks), followed with 12xPaclitaxel (80 mg/m² i.v./week)+ (Trastusumab 6mg/kg+420 mg Pertuzumab i.v./3 weeks)	33
**18**	7xPaclitaxel (80 mg/m² i.v./week)	67
**19**	18x nab-paclitaxel (80 mg/m² i.v./week)	50
**20**	18x nab-paclataxel (80 mg/m² i.v./week)+Bevacizumab (15 mg/kg i.v./3 weeks)	100
**21**	6xPaclitaxel (80 mg/m² i.v./week)+Bevacizumab (15 mg/kg i.v./3 weeks)	33
**22**	2xPaclitaxel (80 mg/m² i.v./week)+Bevacizumab (15 mg/kg i.v./3 weeks)	33
**23**	3xETC (Epirubicin 150 mg/m²+Paclitaxel 225 mg/m²+Cyclophosphamide 2000mg/μ2) i.v./2 weeks	100
**24**	3xETC (Epirubicin 150 mg/m²+Paclitaxel 225 mg/m²+Cyclophosphamide 2000mg/μ2) i.v./2 weeks	75
**25**	3xETC (Epirubicin 150 mg/m²+Paclitaxel 225 mg/m²+Cyclophosphamide 2000mg/μ2) i.v./2 weeks	63
**26**	3xETC (Epirubicin 150 mg/m²+Paclitaxel 225 mg/m²+Cyclophosphamide 2000mg/μ2) i.v./2 weeks	63
**27**	4x EC (Epirubicin 90 mg/m²+Cyclophoshamide 600 mg/m²) i.v./3 weeks, followed with 4x Docetaxel (75 mg/m² i.v./3 weeks)	63
**28**	4x EC (Epirubicin 90 mg/m²+Cyclophoshamide 600 mg/m²) i.v./3 weeks, followed with 4x Docetaxel (75 mg/m² i.v./3 weeks)	75
**29**	4x EC (Epirubicin 90 mg/m²+Cyclophoshamide 600 mg/m²) i.v./3 weeks, followed with 4x Docetaxel (75 mg/m² i.v./3 weeks)	0
**30**	4x EC (Epirubicin 90 mg/m²+Cyclophoshamide 600 mg/m²) i.v./3 weeks, followed with 4x Docetaxel (75 mg/m² i.v./3 weeks)	25

Nr.	Therapy	Dkk1-IRS reduction (%)
**31**	4x EC (Epirubicin 90 mg/m²+Cyclophoshamide 600 mg/m²) i.v./3 weeks, followed with 4x Docetaxel (75 mg/m² i.v./3 weeks)	100
**32**	4x EC (Epirubicin 90 mg/m²+Cyclophoshamide 600 mg/m²) i.v./3 weeks, followed with 4x Docetaxel (75 mg/m² i.v./3 weeks)	75
**33**	4x EC (Epirubicin 90 mg/m²+Cyclophoshamide 600 mg/m²) i.v./3 weeks, followed with 4x Docetaxel (75 mg/m² i.v./3 weeks)	33
**34**	4x EC (Epirubicin 90 mg/m²+Cyclophoshamide 600 mg/m²) i.v./3 weeks, followed with 4x Docetaxel (75 mg/m² i.v./3 weeks)	0
**35**	4x EC (Epirubicin 90 mg/m²+Cyclophoshamide 600 mg/m²) i.v./3 weeks, followed with 4x Docetaxel (75 mg/m² i.v./3 weeks)	78
**36**	4x EC (Epirubicin 90 mg/m²+Cyclophoshamide 600 mg/m²) i.v./3 weeks, followed with 4x Docetaxel (75 mg/m² i.v./3 weeks)	0
**37**	4x EC (Epirubicin 90 mg/m²+Cyclophoshamide 600 mg/m²) i.v./3 weeks, followed with 4x Docetaxel (75 mg/m² i.v./3 weeks)	25
**38**	4x EC (Epirubicin 90 mg/m²+Cyclophoshamide 600 mg/m²) i.v./3 weeks, followed with 4x Docetaxel (75 mg/m² i.v./3 weeks)	75
**39**	4x EC (Epirubicin 90 mg/m²+Cyclophoshamide 600 mg/m²) i.v./3 weeks, followed with 2x Docetaxel (75 mg/m² i.v./3 weeks)	0
**40**	4x EC (Epirubicin 90 mg/m²+Cyclophoshamide 600 mg/m² i.v./3 weeks), followed with 4x Docetaxel (75 mg/m² i.v./3 weeks)+Trastusumab	0
**41**	4x EC (Epirubicin 90 mg/m²+Cyclophoshamide 600 mg/m² i.v./3 weeks), followed with 4x Docetaxel (75 mg/m² i.v./3 weeks)+Trastusumab	67
**42**	9xDocetaxel (75 mg/m² i.v./3 weeks)+(Trastusumab 6mg/kg+Pertuzumab 420 mg i.v./3 weeks)	50
**43**	6xTCH (Docetaxel 75mg/m²+Carboplatin AUC 6+Trastuzumab 6 mg/kg+Pertuzumab 420 mg) i.v./3 weeks	50
**44**	6xTCH (Docetaxel 75mg/m²+Carboplatin AUC 6+Trastuzumab 6 mg/kg+Pertuzumab 420 mg) i.v./3 weeks	83
**45**	6xTAC (Taxotere 75 mg/m²+Adriamycin 50 mg/m²+Cyclophosphamide 500 mg/m²) i.v./3 weeks	50
**46**	6xTAC (Taxotere 75 mg/m²+Adriamycin 50 mg/m²+Cyclophosphamide 500 mg/m²) i.v./3 weeks	100
**47**	6xTAC (Taxotere 75 mg/m²+Adriamycin 50 mg/m²+Cyclophosphamide 500 mg/m²) i.v./3 weeks	0
**48**	6xTAC (Taxotere 75 mg/m²+Adriamycin 50 mg/m²+Cyclophosphamide 500 mg/m²) i.v./3 weeks	33
**49**	2xTAC (Taxotere 75 mg/m²+Adriamycin 50 mg/m²+Cyclophosphamide 500 mg/m²) i.v./3 weeks	56
**50**	4xEC (Epirubicin 90 mg/m²+Cyclophoshamide 600 mg/m² i.v./3 weeks)	33

All protocols were applied according to the German guideline on early detection, diagnosis, treatment of breast cancer [4]. i.v.: Intravenous. AUC: Area under the free carboplatin plasma concentration versus time curve.
