# Supplementary material for: Dkk1 as a Prognostic Marker for Neoadjuvant Chemotherapy Response in Breast Cancer Patients
Source: Cancers (Basel). 2024 Jan 18;16(2):419. doi: 10.3390/cancers16020419 (PMC10814026; doi:10.3390/cancers16020419)
Supplement: Supplementary file 1 [file cancers-16-00419-s001.zip › Supplementary Table S4.pdf]

Supplementary Table S4. The correlation between percentage of Dkk1-IRS reduction and NACT characteristics

	**Group 1**	**Group 2**	**Group 3**	***p* value**
	**Reduction percentage <50%**	**Reduction percentage 50–75%**	**Reduction percentage >75%**	
**Completed therapy**				
Yes	20 (40%)	20 (40%)	10 (20%)	0.410
No	3 (20%)	8 (53%)	4 (27%)	
***Therapy protocol**				
All therapy included Paclitaxel	11 (42%)	9 (35%)	6 (23%)	0.652
All therapies included Docetaxel	13 (57%)	6 (26%)	4 (17%)	

* Depending on whether Paclitaxel or Docetaxel was used as a taxane agent, two groups of therapy protocols were created. Data are presented as number (N) and percentage (%). *p* value is calculated using the chi-square test.
